# Compensating thickness effects in micro X-ray fluorescence spectroscopy using integrated optical microscopy for thickness determination of soft matter block copolymer membranes

**DOI:** 10.1007/s00216-025-06292-2

**Published:** 2025-12-19

**Authors:** Riccarda Müller, Leon Weckenmann, Nigar Aslanova, Yesleen Gupta, Felix H. Schacher, Carsten Streb, Kerstin Leopold

**Affiliations:** 1 Institute of Analytical and Bioanalytical Chemistry (IABC), Ulm University, Albert-Einstein-Allee 11, 89081 Ulm, Germany; 2 Institute of Organic Chemistry and Macromolecular Chemistry (IOMC), Friedrich Schiller University Jena, Lessingstraße 8, 07743 Jena, Germany; 3 Department of Chemistry, Johannes Gutenberg University Mainz, Duesbergweg 10-14, 55128 Mainz, Germany; 4 Jena Center for Soft Matter (JCSM), Friedrich Schiller University Jena, Philosophenweg 7, 07743 Jena, Germany; 5 Center for Energy and Environmental Chemistry Jena (CEEC Jena), Philosophenweg 7a, 07743 Jena, Germany; 6 Helmholtz Institute for Polymers in Energy Applications Jena (HIPOLE Jena), Lessingstraße 12-14, 07743 Jena, Germany

**Keywords:** Block copolymer membranes, Two-dimensional micro X-ray fluorescence spectroscopy, Thickness determination, Thickness compensation, Elemental mapping, Functional soft matter material for photocatalysis

## Abstract

A non-invasive method using the integrated optical microscope of a laboratory-based 2-dimensional micro X-ray fluorescence spectroscopy (2D µXRF) instrument to determine the thickness of soft matter samples has been successfully developed, validated, and applied. This easy-to-use method is applicable to soft matrices in a thickness range from 25 to 1000 µm. The main advantage of this method is that thickness determination is directly related to the physical thickness of the sample, rather than its optical thickness, and it does not affect the sample structure, i.e., it does not require any drying or embedding. Elemental composition and distribution analysis by 2D µXRF can be performed on the same sample specimen, using the same setup directly before or after thickness determination. Knowledge of the thickness of different samples can be used to normalize elemental intensities to compensate for the mass-thickness effect, enabling reliable comparison of samples of different thicknesses. By systematically probing several points on a sample surface, this approach can also be used to correct the elemental intensity of unevenly thick soft matter samples. The aspect of correcting element intensities based on the determined thickness should enable direct quantification of element contents using µXRF and external calibration in future work.

**Graphical Abstract:**

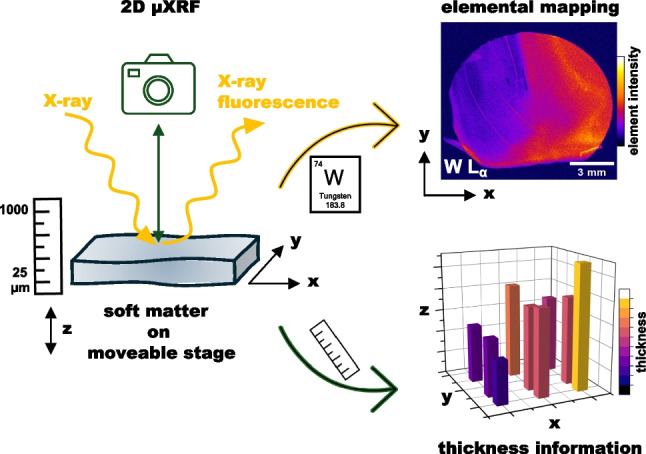

**Supplementary Information:**

The online version contains supplementary material available at 10.1007/s00216-025-06292-2.

## Introduction

In recent years, two-dimensional micro X-ray fluorescence spectrometry (2D µXRF) in benchtop setups has become a widely used analysis technique for quickly and easily characterizing lateral element distribution of relatively flat samples. A focused X-ray beam of a few micrometers is directed onto the sample, which is placed on a movable stage. Scanning the sample and detecting its X-ray fluorescence produces 2D elemental maps. Depending on the configuration of the excitation source, X-ray optics and detector, it is possible to achieve semi-quantitative information on elements ranging in atomic number from 5 (boron) to 92 (uranium) with a lateral resolution of a few micrometers [1–3]. In comparison to other laboratory-based elemental mapping techniques such as laser ablation inductively coupled plasma mass spectrometry (LA-ICP-MS) [4], secondary ion mass spectrometry (SIMS) [5] or scanning electron microscopy with energy dispersive X-ray spectroscopy (SEM/EDX) [6], 2D µXRF has several advantages [7]. It requires minimal or no sample preparation, is non-destructive and provides multi-element analysis over a broad concentration and element range. Moreover, it can quickly analyze representative, relatively large sample areas (~ 100 cm^2^) using cost-efficient instrumentation. Common application fields of laboratory-based 2D µXRF include materials science [8–10], geology [11, 12], environmental sciences [13, 14], archaeology [15, 16], and forensics [17]. Soft materials that deform easily under the influence of external stresses, electric or magnetic fields, or thermal fluctuations [18], such as liquids, soft polymers, foams, gels, or soft biological materials, are less commonly investigated by µXRF. Nevertheless, there are several recent examples showing that µXRF is a useful alternative technique for elemental mapping of e.g. biological tissues [4, 19–22] or gels [23]. However, soft matter matrices often consist mainly of so-called 'dark matrix' elements such as H, C, N, and O, which do not exhibit sufficient X-ray fluorescence intensity to be detected [24]. Additionally, the primary beam penetrates relatively deeply below the surface of the sample, yielding a depth-averaged XRF signal for visible elements [25]. This makes interpreting the corresponding µXRF spectra of 'visible' elements more challenging with regard to quantification. Hence, even for semi-quantitative comparison µXRF data of samples of the same type, it is crucial to know the thickness of each individual sample. Moreover, (absolute) quantification is generally more difficult in µXRF than in XRF due to the heterogeneous nature of the materials investigated. In general, quantification using XRF can be achieved using external calibration standards or standardless computational approaches such as the fundamental parameter (FP) method and the emission-transmission (ET) method [26]. In FP method, the assumptions underlying the mathematical calculations, known as the *Sherman* equation, require knowledge of the theoretical composition of the sample. To simplify the calculations, it is usually assumed that the sample is infinitely thick [27], although suggestions have been made as to how to modify the calculations for thin film samples [28]. Hence, applying the FP method is particularly challenging when the main components of the samples are dark matrix, the sample cannot be considered infinitely thick, and low-energy fluorescence is to be detected [24]. An alternative is the ET method, which can be used to correct for matrix absorption effects and therefore compensate for differences in thickness of homogeneous samples [29]. For µXRF applications involving heterogeneous samples, such as biological tissues, it is not feasible and external calibration using gelatine standards sufficiently similar to the samples has been proposed [4]. To achieve this, sections with precisely defined, identical slice thicknesses were prepared for the external standards and the tissue sample. In conclusion, calibrating µXRF for the quantification of visible elements in dark matrix samples requires either the preparation of known sections or knowledge of the sample's thickness.

With regard to dark matrix samples, we are particularly interested in characterizing soft matter materials that are used in photocatalysis, i.e., samples in which light-driven molecular catalysts and photosensitizers are embedded in a soft matter matrix and enable photoreactions involving oxidation and/or reduction [30]. The non-destructive nature of µXRF analysis is a major advantage in the process of developing and investigating such materials, as it enables multi-approach analysis to be performed on the same specimen, allowing clear unbiased conclusions to be drawn about element distribution, sample uniformity, and eventual leaching, thereby paving the way to structure–property analysis [31]. Examples of such material are so-called ‘POMbranes’, in which polyoxometalates (POMs) are embedded in a nanoporous block copolymer membrane [32]. The membranes consist of polystyrene-*block*-poly(2-(dimethylamino)ethyl methacrylate) (PS-*b*-PDMAEMA), a block copolymer with a hydrophobic (PS) and a hydrophilic (PDMAEMA) block. Phase separation between these two blocks creates a nanostructured, porous morphology [33]. The membranes are prepared using the non-solvent induced phase separation (NIPS) process, with films cast onto a substrate using the doctor blade technique in a climate chamber. The porosity can be tuned by selection of the proportion of DMAEMA to PS or by altering the solvent ratio of DMF to THF used for casting. After casting, membranes form by exposure to air ('open time'), after which they are quickly immersed in a coagulation water bath and kept in water for storage. Selecting the gate height of the doctor blade allows different thicknesses between 50 µm and 200 µm to be prepared, although the gate height and the thickness of the membrane are not identical due to the formation of the porous structure. POMs can be immobilized in these membranes due to attractive electrostatic interactions. POMs are polynuclear transition metal oxide anions that are renowned for their unique redox properties, making them ideal for use as molecular catalysts in redox catalysis [34, 35]. Thorough characterization of POMbranes is crucial for evaluating their catalytic performance, molecule-in-matrix stability and homogeneity of the material. Recently, we demonstrated the feasibility of using 2D µXRF to investigate catalyst and photosensitiser distribution in a POMbrane [31]. In this study it was found that preparation of membranes of different thicknesses results in different catalyst loadings, leaching behavior, and catalytic activity under irradiation. Thicker membranes exhibited lower catalyst loading and prolonged catalytic activity, as well as reduced leaching. Furthermore, it was observed that refilling the pores of the membranes with water after drying resulted in a change in thickness. Therefore, as the nanoporous structure of the membranes is water-filled in both their native and active forms, it is best to characterize them directly without altering their structure through sample preparation. Furthermore, to avoid misinterpreting µXRF spectra when comparing (semi-)quantitative information of different samples, it is crucial to know the thicknesses of the active membranes.

Determining the thickness of soft matter materials is challenging because conventional methods, such as micrometers, vernier callipers or thickness gauges, are unsuitable due to the risk of compressing the sample when force is applied [36–38]. On the other hand, electron microscopy [39, 40] and histomorphometry [41] require preparation steps such as drying or freezing, embedding and sectioning. As detailed above, this renders the membranes unusable for further application or subsequent analysis of element distribution. Though non-invasive techniques such as optical coherence tomography [42] and optical profilometry [43, 44] have been suggested in the field of (bio)medical research and application, these methods require costly instrumentation and are not sufficiently compatible with the investigation of the nanoporous membranes investigated here. The objective of this study was therefore to develop a non-destructive, direct method for reliably and reproducibly determining the thickness of active, water-filled, nanoporous membranes, thereby providing reliable data for an external calibration approach in µXRF quantification. Ideally, the thickness determination method would have the potential to provide lateral resolution in order to account for non-homogeneous samples, thereby enabling correlation with elemental intensity maps detected by laboratory-based µXRF.

## Materials and methods

### µXRF instrumentation and measurements

M4 Tornado µXRF instrument (Bruker Nano GmbH in Berlin, Germany) equipped with Rh X-ray tube operated at maximum power (50 kV, 600 µA), a X-Y-Z stage (max. speed: 100 mm s^−1^, max. acceleration 200 mm s^−2^, traversing range: 200 × 160x120 mm; mapping range: 190 × 160 mm) driven by direct current (DC) motor with encoders and charge coupled device (CCD) color cameras with dual magnification (camera 1: 10x, field of view: 14 × 11 mm^2^; camera 2: 100x, 1.4 × 1.1 mm^2^) is used. The polychromatic beam is focused by polycapillary lenses to a spot of around 25 µm diameter. The resulting X-ray fluorescence is detected by an XFlash 430 PA detector with an active area of 30 mm^2^ and a resolution of < 145 eV at Mn K_α_. Measurements were carried out under ambient air conditions and no filters were applied. For elemental mappings, a dwell time of 10 ms per pixel was used and point measurements were carried out with a live time of 10 s.

### Height measurements using X–Y-Z stage of µXRF

A self-developed 3D-printed sample holder made of polylactide (PLA) was used for all measurements (see Supporting Information, SI; Figure S1). This consists of a lower part with a rectangular frame cut-out. A polyvinyl (PV) film or acrylic glass plate (3 mm) can be placed on top of this, onto which the sample is placed. There is no further material underneath to minimize scattering. To protect the sample from drying out and to keep it planar, a thin Ultralene® (4 µm) window foil (Cole-Parmer SamplePrep, Metuchen NJ, USA) with an upper frame is stretched over it. The markings for the height measurement were then painted onto the Ultralene® film. For this purpose, a silver metallic (Faber Castell, Stein, Germany) and a blue Lumocolor permanent (Staedtler, Nürnberg, Germany) foil pen were used. For gelatine film and membrane samples only silver mark was used. The sample holder was placed in a cut-out mold on the X-Y-Z-stage to ensure a fixed position. The stage is driven by direct current (DC) motors with encoders. The encoder is a sensor that provides feedback on the motor's position and speed, enabling precise control and monitoring of the motor's rotational movement. This combination allows for closed-loop control, where the system can adjust the motor's behavior based on real-time feedback from the encoder. Precision in X-, Y- direction is 0.01 mm and in Z-direction 0.001 mm. For height measurements using the stage, a point was selected for each marking and the X- and Y-coordinates were saved. After changing the film, the position was returned to the same point, which ensures that the focus can then be adjusted at the same point again. For the height measurement, the focus on the markings was set manually using the CCD camera with the 10 × magnification. Then an initial stage height (${z}_{start}$) 50 to 100 µm below the focus height was selected and the autofocus function was activated. After noting the height ${z}_{i}$, the stage was manually set again to the initial height. For each marking, this procedure was repeated at least three times. For evaluation of reproducibility of auto focus versus manual focus the calibration foil with a thickness 79.1 µm (Elcometer Limited, Manchester, UK) was measured ten times. (See also SI Figure S2 for a schematic representation of the procedure.)

### Measurement of attenuation of Cr Kα X-ray fluorescence

For the measurements of attenuation of X-ray fluorescence of an underlying metal foil by the membrane, a chromium-sputtered PV foil was placed into the sample holder instead of the acrylic glass slide. Chromium (Cr) thin film was deposited in a glove box using RF magnetron sputtering device PROvap 4G (M. Braun Inertgas-Systeme GmbH, Garching, Germany) with a Cr target (Evochem Advanced Materials GmbH, Offenbach, Germany). Argon was used as sputtering gas at room temperature at a working pressure of 1*10^–2^ mbar with an RF power of 50 W for Cr for a deposition time of 15 min.

### Contrast evaluation

#### Markings

The ink in the silver pen contains small metal particles consisting of mainly Al and traces of Fe, Ti, Cr, Zn and Ga (as detected by TXRF, see SI Table S1), which create a textured mark on the film. The blue pen contains mainly Cu, S, and Cl and leaves a smooth, colored mark on the foil. Both pens can be used to create shades of color, depending on how much ink is applied to the film.

#### Image evaluation

Images obtained by the CCD camera of the µXRF instrument are evaluated using *Fiji* software (1.54f 13/02/2025) [45]. For contrast evaluation grey value histograms of the images were generated, and the standard deviation was assessed. In this case, the standard deviation is equal to the grey value spread.

### Thickness reference measurement by light microscopy and micrometer

#### Light microscopy

*Axio Imager.M1m* light microscope (Carl Zeiss AG, Oberkochen, Germany) was used to measure the gelatine and membrane cross-sections. The analysis was performed using a lens at 10 × magnification (EC Epiplan-Neofluar, Carl Zeiss AG, Oberkochen, Germany) and different contrast managers (bright field, dark field, and differential interference contrast in circularly polarized light), depending on where the edges of the sample could be viewed better. The thickness of the membrane was measured using *Axio Vision* Software (AxioVS 40 × 64 V 4.9.1.0, Carl Zeiss AG, Oberkochen, Germany). A circular membrane cut-out of an unloaded membrane was cut through the center using a scalpel. The membrane was fixed between two microscope glass slides with cut edges (VWR International bvba, Leuven, Belgium) with the sides of the slides flush with the cut edge of the membrane.

#### Micrometer

A digital *Micrometer IP54* (Mahr GmbH Helios-Preisser Vertrieb, Gammertingen, Germany) with a resolution of 0.001 mm was used to measure the thickness of the gelatine film samples.

### Calibration foils

For validation of the proposed method for thickness determination different calibration foils with certified thickness were used, ranging from 21.9 µm to 1000 µm. The exact thickness and manufacturer information can be found in Table 1.

**Table 1:** Manufacturer information and thicknesses of the used calibration foils

*Company*	*Thickness, µm*
Elcometer Limited, Manchester, UK	23.9, 50.8, 79.1, 126, 172.9, 249, 486
List-Magnetik GmbH, Leinfelden-Echterdingen, Germany	30, 98, 300, 1000
KARL DEUTSCH Prüf- und Messgerätebau GmbH + Co KG, Wuppertal, Germany	21.9, 46.1, 94.7, 189.3, 739

### Preparation of gelatine foil samples

Gelatine foil was prepared as described by *Andreuccetti *et al. [46]. Therefore 10 wt% gelatine solution was prepared from 1 g gelatine powder and 8 mL ultrapure water (UPW). After 15 min swelling the gelatine solution was homogenized at 60 °C for 10 min. The hot gelatine solution was poured onto an acrylic glass plate and dried overnight at room temperature. The resulting film was then removed from the plate. Thickness of the gelatine foil was measured minimum five times per piece using a digital micrometer. The thicknesses were also determined via the cross-section on one of the sides using an optical microscope.

### POMbrane samples

Membrane and POMbrane samples are made of polystyrene-*block*-poly(2-(dimethylamino)ethyl methacrylate) (PS-*b*-PDMAEMA). The hydrophilic PDMAEMA units can be positively charged, and the polystyrene block gives the membrane stability. Functionalized with metal-based catalyst (CAT), like polyoxometalates (POM), and photosensitizer (PS) complexes, these nanoporous membranes can be used in light-driven, artificial photosynthesis to split water into hydrogen and/or oxygen. More detailed information on POM synthesis, membrane preparation and characterization can be found in the Supporting Information*.*

The functionalization of the membranes using POM was carried out according to the procedure reported by *Kund *et al*.* [31] using 0.64 mmol L^−1^ of [Co_4_(H_2_O)_2_(PW_9_O_34_)_2_]^10−^ POM in 20 mL UPW. After immersing the membrane for 24 h in a round-bottom flask with a protective polyamide mesh layer, the resulting POMbranes were stored in UPW until usage.

## Results and discussion

### Principle of thickness determination using integrated optical microscopy

**Fig. 1:**
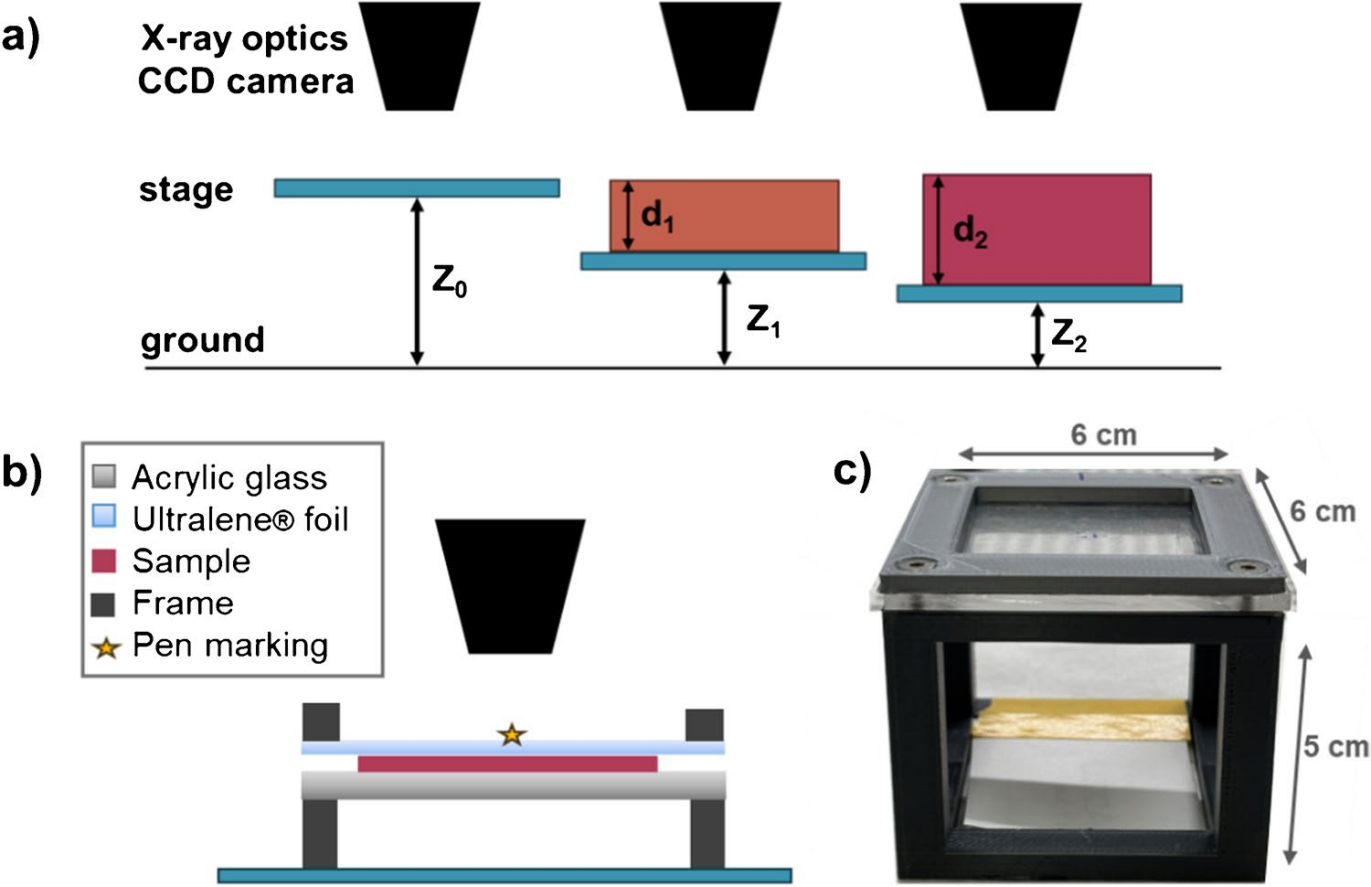
**a)** Schematic illustration of thickness determination using integrated optical microscopy of µXRF instrument’s X-Y-Z stage. **b)** Schematic of side few of sample holder and **c)** picture of sample holder.

The developed method for thickness determination of soft matter samples, i.e., nanoporous water-filled polymer membranes, utilizes the stage position and the autofocus of the CCD camera of the tabletop µXRF instrument as illustrated schematically in Fig. 1a. Initially, a 3D-printed sample holder was designed and constructed in-house, incorporating a rigid acrylic glass plate on which the sample can be placed and an X-ray transparent foil that covers the thin water-filled porous membrane sample (see Fig. 1b and c). The function of the covering foil is to prevent the samples from drying out during the measurement process.

The measurement procedure is straightforward as outlined in the following: Initially, the CCD camera focuses on the upper surface of the empty sample holder, subsequently on the top of a respective sample. It is evident that the thickness of the specimen directly correlates with the stage's Z-movement to achieve optimum focus. The thickness ${d}_{i}$ of the sample i at a defined sample spot can be determined by the following Eq. (1), provided that the coordinates of the stage have been saved:

1 $${d}_{i}={z}_{0}- {z}_{i}$$

where ${z}_{0}$ is a position on the Z-coordinate without sample and ${z}_{i}$ the height of the stage with sample i.

The following three aspects were considered in regard to the feasibility, optimisation and validation of the proposed method for thickness measurement, in the following referred to as ‘integrated optical microscopy stage method’, short ‘IOM stage method’:

A. (How) Can the focus be determined highly reproducibly at different spots and on different samples?
B. Is the Z-movement of the built-in stage, when used with the designed sample holder, accurate and reproducible enough to measure thicknesses of a few hundred µm with an acceptable margin of error?
C. Is the proposed method applicable to water-filled (nano)porous soft matter matrices?

### Reproduceable determination of focus

The focus of the tabletop µXRF instrument used here can be set manually or automatically. Preliminary tests (with n = 10) demonstrated that manual focusing is less accurate, resulting in significantly higher combined relative standard deviation of approximately 72% compared to 12% when employing autofocus. The autofocus is achieved through the identification of the most significant contrast: Initially, a manual coarse adjustment is executed at 10 times magnification of a CCD camera, and subsequently, to achieve fine focusing the autofocus is used at 100 times magnification. Consequently, the autofocus function was utilized to initiate the focusing process, commencing from a z-position that was evidently lower than the actual sample height.

In order to further optimize the autofocusing process by identifying the highest contrast, a set of experiments was conducted. The contrast is evaluated by determining the grey value spread [47]. Namely, the greater the grey value spread, the better the image is in focus and the sharper the picture (see exemplary images in Supporting Information (SI), Figure S5). Accordingly, different LED lighting intensities (low: 11 a.u.; high: 30 a.u.) within the sample chamber of the µXRF instrument were tested in conjunction with pen markings of two distinct colors, i.e., silver and blue, on the coverage film of simulated samples of diverse colouration (white, grey and black paper). It is hypothesized that optimizing these parameters to obtain high contrast will allow for more precise autofocus, even on unicoloured samples, thereby impacting the accuracy of thickness measurements.

**Fig. 2:**
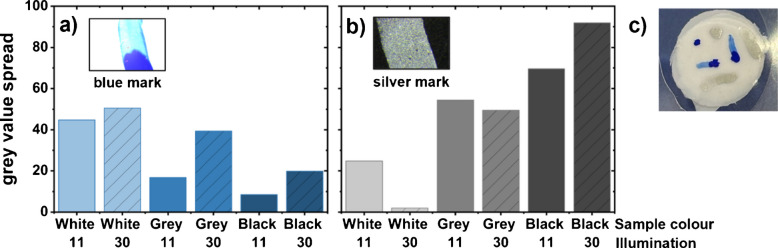
Grey value spread for **a**) blue and **b**) silver marks on coverage film on white, grey and black simulated sample using high (30 a.u.) and low (11 a.u.) lighting intensity. **c**) Photo of blue and silver marks on exemplary simulated sample

Both pens can be used to create shades of color, depending on how much ink is applied to the film (see Fig. 2c). The elemental composition of the inks was examined by using total reflection X-ray fluorescence spectrometry (TXRF; see SI, Table S1) to exclude any potential overlap between existing elements and future unknown analytes.

The results presented in Fig. 2 show that both marking colors result in suitable grey value spreads for autofocusing. However, the silver markings generally exhibit higher contrasts except for the white samples. Moreover, our hypothesis that the precision of thickness determination would be better for a higher grey value spread was confirmed when we evaluated replicate autofocusing for the highest and lowest grey value spreads (see SI, Table S2). The obtained standard deviations of the Z-coordinate represent the depth of focus of the proposed method and range from ± 3 µm for a grey value spread of 94.52 ± 0.01 (n = 10; silver marking, black sample, high illumination of 30 a.u.) to ± 6 µm for a value of 2.22 ± 0.01 (n = 10; silver marking, white sample, high illumination of 30 a.u.). Considering that the specimen range in thickness from fifty to a few hundred micrometers, a variation of 3 µm or 6 µm is reasonable. In conclusion, the respective marking color can be selected methodically for analyzing real samples of various colors.

### Verification of thickness determination

**Fig. 3:**
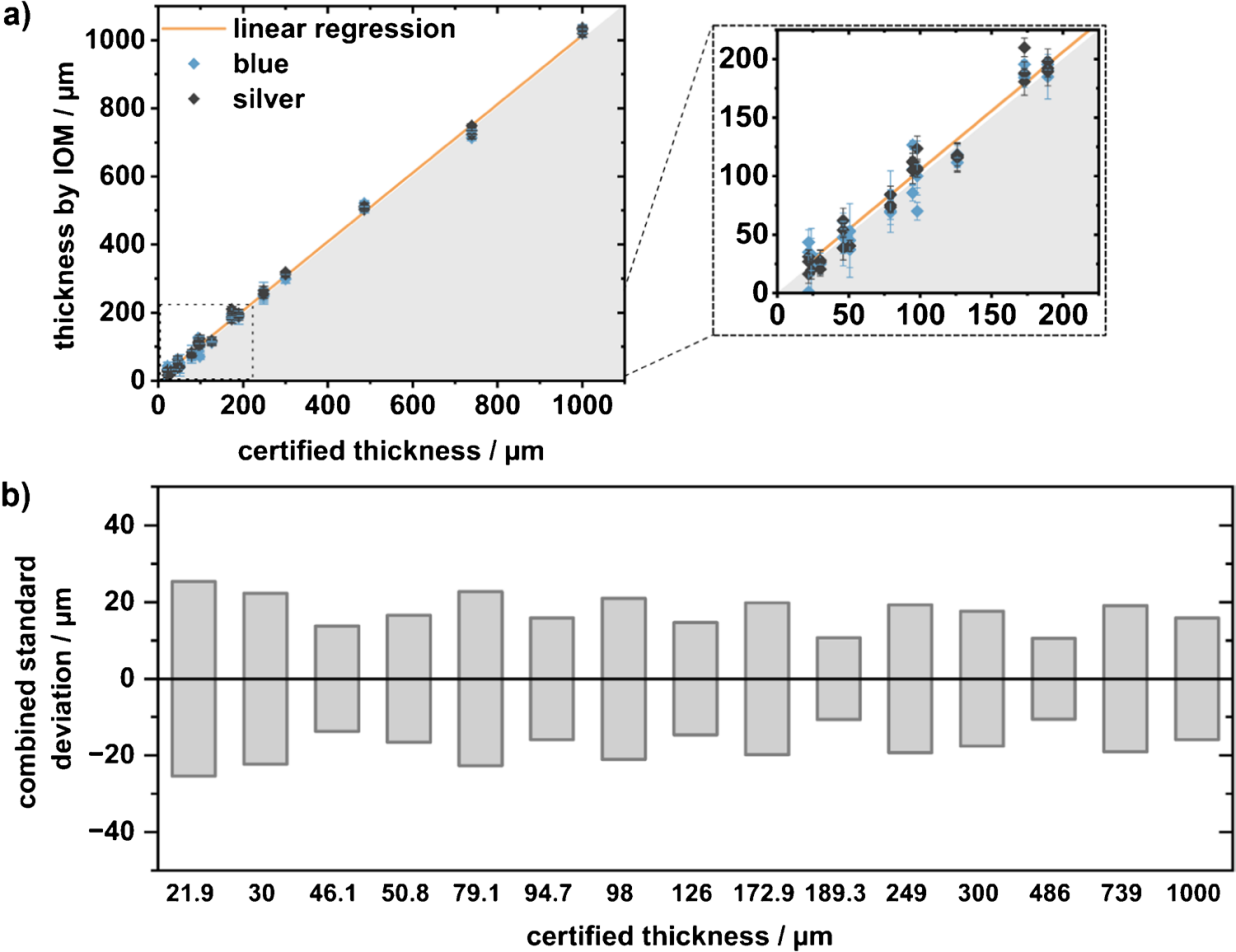
**a**) Recovery function obtained for thicknesses determination of calibration foils using IOM stage method and differently colored marks. Recovery function: ${\boldsymbol{y}}\boldsymbol{ }=\boldsymbol{ }\left(1.01\boldsymbol{ }\pm \boldsymbol{ }0.01\right){\boldsymbol{x}}+\boldsymbol{ }(4.08\boldsymbol{ }\pm \boldsymbol{ }2.09)$. Error bars reflect combined standard deviations of replicate measurements with *n* = 3. **b)** Combined standard deviations for thickness determination at 6 different positions on the calibration foils. Number of replicate measurements at one position was *n* = 3

Calibration foils with certified thicknesses ranging from 21.9 to 1,000 µm were used to verify the thickness measurements obtained using the IOM stage method in accordance with Eq. (1). In Fig. 3 the values obtained for the determined thicknesses are plotted against the certified values. The corresponding recovery function, which includes all data points, results in a recovery rate of 101 ± 1% (*n* = 3; *N* = 91; *P* = 95%). Evaluation of the two individual marking colors reveals no significant difference in accuracy, but slightly lower precision when using the blue marker, namely a relative residual standard deviation of 6% compared to 5% for the silver marker.

Repeated measurements taken at 6 different positions on a calibration foil produced consistent results, with an arithmetic mean of the combined standard deviations of ± 18 µm and a maximum deviation of ± 25 µm (see Fig. 3b). In conclusion, the precision achieved by Z-movement of the built-in stage in combination with our designed sample holder is sufficiently accurate for determining the thicknesses of solid, uniform samples in range from about 50 µm to 1000 µm providing an uncertainty of a few 10 µm.

The next intermediate step was to analyze self-prepared gelatine films to evaluate the IOM stage method's suitability for determining the thickness of dark matter materials. These films exhibit a combination of flexibility and rigidity, which makes them less uniform than the calibration foils with defects such as surface scratches and air inclusions. However, thickness can still be determined using a micrometer and a light microscope as reference methods. Furthermore, the gelatine pieces are smaller than the calibration foils, meaning they only sit in the middle of the sample holder rather than between the frames. The thickness of six gelatine foil samples was measured at multiple points on the films using the IOM stage method with blue and silver markings, as well as a micrometer, and at one of the edges using a light microscope. The results obtained ranged from 94 µm to 111 µm, and were except for two outliers all consistent with one another (see SI, Figure S6a). The observed outliers - i.e., slightly higher values observed by the IOM stage method - can be attributed to the challenges encountered during sample preparation, namely the inserting of the sample into the holder can be problematic, as the gelatine foil pieces tend to repel the carrier and cover foil. In order to verify accuracy of thickness determination by the IOM stage method over a greater thickness range, up to four films were arranged in a superimposed configuration and measured using a micrometer for a reference (SI, Figure S6b). To minimize repulsion, the individual layers were carefully pressed together after the samples were placed in the measurement setup. Following this optimized sample preparation, the obtained thickness values are reasonably consistent (recovery: 90 ± 4%), confirming the feasibility of using the IOM stage method for soft matter materials within the tested size range of 100 to 400 µm.

### Application to water-filled nanoporous polymer membranes

Finally, the IOM stage method was applied to determine the thickness of a real sample: a nanoporous block copolymer membrane functionalised with a POM-based catalyst and a Ru-based photosensitiser. These membranes are based on polystyrene-*block*-poly(2-(dimethylamino)ethyl methacrylate) (PS-*b*-PDMAEMA), and were prepared using the doctor blade technique, followed by storage in water [32].

**Fig. 4:**
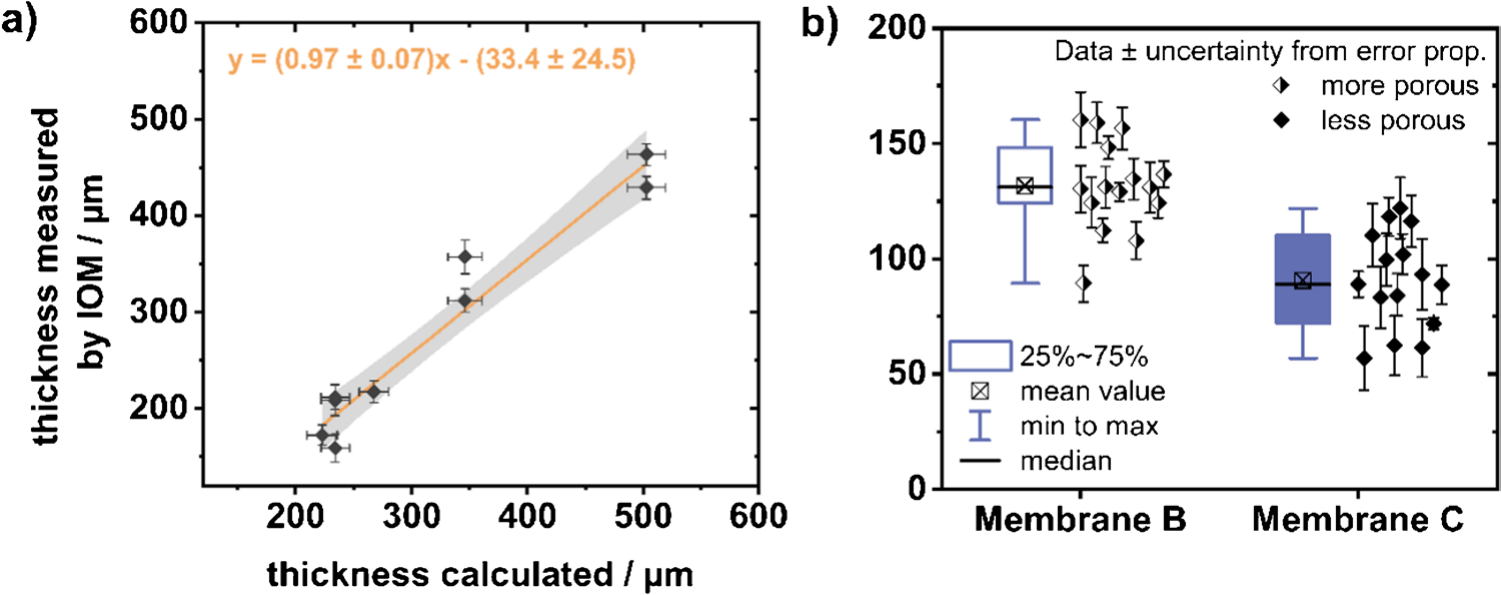
**a)** Recovery function obtained by determination of thickness of membrane stacks using IOM stage method compared to calculated thickness derived from the sum of individual thickness of membrane pieces (Membrane A). X-error bars represent uncertainty as derived from *Gaussian* error propagation of replicate measurements with *N* = 15 and *P* = 95%; Y-error bars represent combined standard deviation of replicate measurements with *n* = 9 and *P* = 95%. **b)** Thickness determined for membranes of the same composition with either higher porosity (Membrane B; half-filled diamonds) or lower porosity (Membrane C; filled diamonds). Error bars represent combined standard deviation of replicate measurements with *n* = 3 and *P* = 95%

First, five circular pieces measuring 38.5 mm^2^ were punched out of the membrane sheet (*Membrane A*) and then measured individually several times using the IOM stage method after six markings had been applied to random positions. Evaluation of the two marking colors revealed significantly higher precision with a mean deviation of ± 13 µm when using the silver marker. The mean thicknesses obtained for the five membrane pieces range between 86 ± 17 µm and 157 ± 7 µm (all values see SI; Table S3). As it is not possible to use a micrometer or light microscope to determine the reference thickness here, the thickness determined by the IOM stage method could only be verified indirectly. To this end, the individual membrane pieces were stacked and their thickness was determined and compared to those calculated from the thicknesses of the individual membranes. Figure 4a shows the recovery function obtained for this experiment (recovery rate: 97 ± 7%; intercept: 33.4 ± 24.5 µm), confirming good agreement between the measured and calculated thicknesses of the stacked membranes, and the absence of proportional systematic error. However, it is evident that the overall precision in this recovery experiment is significantly lower than in the preceding experiments. This can be explained by the difficulties involved in the stacking of the membrane pieces in the sample holder. Attraction or repulsion may occur during stacking, given that the membranes are positively charged and the thickness of the water films between them may vary. These difficulties would not arise if there was a thicker membrane instead of a stack of thinner membranes.

Therefore, the next step was to investigate two membranes with identical polymeric compositions but distinct porosities, which were achieved by using different solvent ratios during the membrane formation process. Five circular pieces measuring 20 mm^2^ were again punched out of the respective block copolymer *Membranes B and C* and silver marking were applied on three random positions before measuring thickness by the IOM stage method. As would be anticipated, *Membrane B,* which is more porous, exhibits a greater average thickness in comparison to *Membrane C*, which is more dense and therefore thinner, as can be seen in Fig. 4b. In addition, both experiments reveal a certain inhomogeneity in the thickness of the membrane sheets themselves. This is why no significant statistical difference between *Membranes B and C* can be proven. The spread of the data points reveals differences in thickness of up to 70 µm for one sample, which are most probably caused by synthesizing and probing different positions, rather than being a result of the analysis method.

To check this hypothesis further, systematic investigations were performed comparing the measured thicknesses under the following variations: a) Same x/y-position, but with the top or bottom of the membrane facing upwards (*Membrane D*); b) after storing the membrane for 11 days (*Membrane D*); and c) at 15 different x/y-positions (*Membrane B*). A modified *Welch's* t-test for unpaired samples (see SI) revealed no significant difference in the determined membrane thickness when the top or bottom of a membrane piece faced upwards. Two measurement series, one performed on the same day and the other after 11 days of storage, also produced no significantly different results when tested using a paired *Student's* t-test (see SI). These results confirm on the one hand that the measurements by the IOM stage method are reproducible and on the other hand that handling and storage conditions do not affect membrane thickness. (The results for these experiments can be found in Table S4 in the SI.) Conversely, the thicknesses determined at 15 randomly selected x/y-positions on *Membrane B* range from 89.33 ± 7.96 µm to 160.33 ± 11.94 µm (see SI, Figure S7) confirming variation in thickness of up to 71 µm.

### Laterally resolved thickness determination

**Fig. 5:**
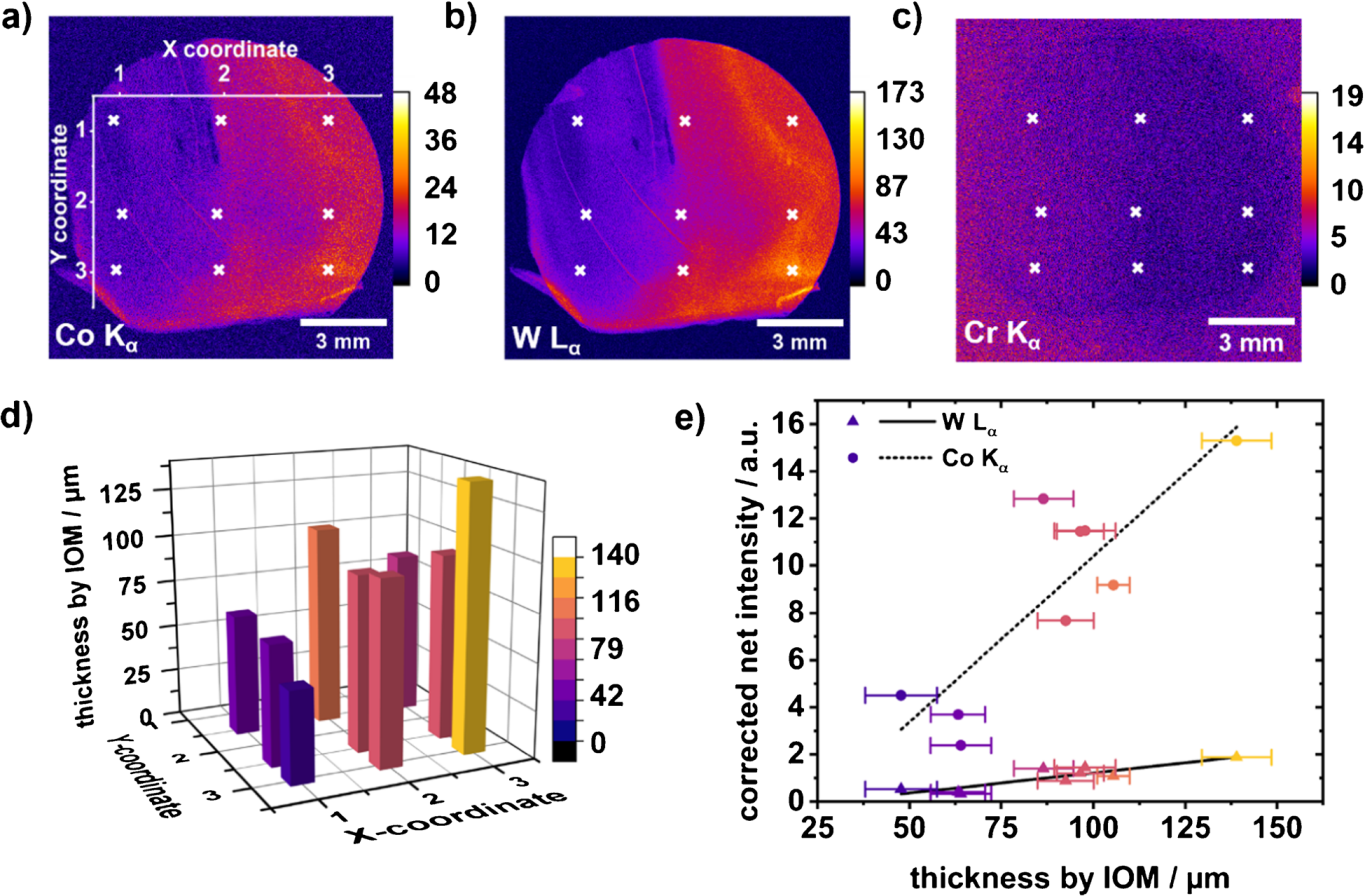
**a)** Element map for Co K_α_ (6.9 keV) and **b)** W L_α_ (8.4 keV) of POM catalyst-loaded *Membrane E,* and indication of selected x/y-positions used for thickness measurement. **c)** Element map of Cr K_α_ (5.4 keV) of sputtered foil beneath *Membrane E* and indication of selected x/y-positions used for thickness measurement*.* **d)** Determined thicknesses of *Membrane E* at nine selected x/y-positions. **e)** Correlation of thickness determined using IOM stage method with C/R ratio corrected Co K_α_ and W L_α_ intensities derived from point spectra. (dashed line Co: ${\boldsymbol{y}}\boldsymbol{ }=\boldsymbol{ }\left(0.140\boldsymbol{ }\pm \boldsymbol{ }0.032\right){\boldsymbol{x}}-\boldsymbol{ }\left(3.653\boldsymbol{ }\pm \boldsymbol{ }2.973\right)$; R^2^ = 0.729; *N* = 9, P = 95%); straight line W: ${\boldsymbol{y}}\boldsymbol{ }=\boldsymbol{ }\left(0.017\boldsymbol{ }\pm \boldsymbol{ }0.004\right){\boldsymbol{x}}-\boldsymbol{ }(0.496\boldsymbol{ }\pm \boldsymbol{ }0.321)$; (R^2^ = 0.774; *N* = 9; P = 95%). X-error bars reflect combined standard deviations of replicate measurements with *n* = 4.)

As the membranes exhibit deviations in thickness greater than the measurement accuracy of the proposed method, we tested whether it was possible to achieve some form of lateral resolution in thickness determination. To this end, a checkered silver marking was applied to the cover film (see SI, Figure S8) and thickness measurements of catalyst-loaded *Membrane E* were taken at the nine crossing points distributed across the membrane, covering a sample area of 38.5 mm^2^. To obtain comparable results, an autofocus area of 1.39 mm^2^ was used, as in the above-described experiments. This corresponds to 80% of the CCD camera's image area at 100-fold magnification. A significantly higher resolution in thickness determination could be achieved by reducing the autofocus area to a minimum of 0.02 mm^2^ using this instrument. However, testing this was beyond the scope of this work. As can be seen in Fig. 5d, a thickness gradient is observed from left to right along the X-coordinate. This finding is consistent with the elemental mapping of the metals W and Co (Fig. 5a and b), assuming that the POM catalyst (here [Co_4_(H_2_O)_2_(PW_9_O_34_)_2_]^10−^) is loaded homogeneously onto the membrane [31]. The highest intensity was detected in position P_9_(3/3), while significantly lower intensities were observed in positions P_1_(1/1) to P_3_(1/3), reflecting less material. Conversely, when the same piece of membrane is placed onto a chromium-coated foil, a lower intensity of Cr K_α_ is observed in the thicker areas of the membrane (see Fig. 5c). For homogeneous, thin samples, it has been suggested that the attenuation of X-ray fluorescence from an underlying material can be used to evaluate thickness in accordance with Lambert–Beer's law [48–50]. The advantage of this approach is that this information is obtained simultaneous to the elemental distribution of the analytes, making the process less time-consuming. However, the material-dependent attenuation coefficient µ must first be shown to be constant across the probed area; in other words, the matrix must be both structurally and chemically homogeneous. Therefore, using attenuation could lead to an inaccurate interpretation of physical thickness for samples with highly heterogeneous element distribution and/or structural inhomogeneities, such as porosity or humidity. This is because, unlike the IOM stage method proposed herein, attenuation is not directly related to the physical thickness of the sample, but rather to its optical thickness.

To enable a more quantitative interpretation of the obtained data, µXRF point measurements were taken at each position where the thickness was determined. Initially, we checked whether the Compton and Rayleigh scattering peaks correlated with the thicknesses of the membrane found, as the correction of elemental intensities using either the Compton scattering intensity or the ratio of Compton to Rayleigh scattering peaks has been suggested in the literature as a means of compensating for the inherent differences in the thicknesses and densities of thin soft matter materials [24, 51]. As can be seen in SI, Figure S9, the scattering intensities and their ratio do not correlate with the thickness found at positions P_1_ to P_9_. However, the Compton-to-Rayleigh scattering ratio (C/R) is not constant across the nine probing positions, which indicates that the membrane's average absorption properties vary slightly. In this context, it should be noted that the sample holder—a 3 mm-thick acrylic glass slide—contributes significantly, albeit evenly, to X-ray scattering and absorption. Therefore, the slight variations observed seem plausible, as they merely reflect differences within the membrane. Since the C/R ratio is proportional to the average atomic number of a sample [27, 52], and since the polymer membrane is highly porous with water-filled pores, variations in pore structure result in different water-to-polymer ratios. Accordingly, the Co and W intensities measured at the nine sampling points were corrected for the C/R ratio before being correlated with the measured thicknesses (see Fig. 5e). The resulting linear regressions are reasonable, with regression coefficients (R^2^) ≥ 0.73 and no intercept, i.e., no intensity at zero thickness, and a linear increase in W and Co with increasing sample thickness. This demonstrates the method's potential for performing thickness scans and providing more reliable quantitative information on element distributions in non-homogeneous, thin soft matter materials.

## Conclusion

In this study, we demonstrated for the first time that the movable sample stage of a 2D µXRF laboratory instrument can be used to determine sample thickness based on differences in z-position. To this end, we designed and built a sample holder that secures the sample at an appropriate height and facilitates reproducible height measurements even after sample changes. To determine the height precisely, the autofocus function of the device, i.e., the built-in CCD camera, is used at 100-fold magnification and to create the greatest possible contrast a mark is applied to a film covering the sample. When examining hard samples, such as calibration foils for thickness determination, a recovery rate of 101 ± 1% was achieved in the size range from 25 to 1000 µm. Examining soft matrices, such as nanoporous polymer membranes is also possible showing only slightly lower precision with a recovery rate of 97 ± 7% in the tested range from 200 to 500 µm. The proposed method has two key advantages: it is non-invasive, avoiding any drying or embedding of the soft matter material, and thickness determination can be carried out using the same µXRF setup as for elemental composition and distribution analysis. Therefore, the thickness determination can be carried out directly before or after the element distribution analysis using µXRF. This means that the results of both measurements can be correlated directly and the determined thickness can be used to compensate for the mass-thickness effect in semi-quantitative analyses. For mapping elements in soft matter samples of uneven thickness, it is also possible to systematically probe several points on the sample surface to correct the element intensity map. To this end, the multi- or auto-point analysis mode of the instrument can be used to autofocus individually on each selected point prior to the XRF measurement. In practice, it should be noted that the samples are sufficiently flat for this purpose, and that thickness determination is then based on a single measurement. Regarding the number of point measurements, a compromise must be found between the time taken and sufficient resolution. In future work, this aspect of element intensity correction will be further developed and incorporated into an external calibration strategy for the direct quantification of elemental contents using µXRF. In addition to its applicability to porous polymer membranes, as demonstrated successfully here, the method has the potential to be applied to other soft matter samples, including gels, aerogels and films, as well as biological and biomedical samples.

## Supplementary Information

Below is the link to the electronic supplementary material.

ESM 1 (DOCX 2.75 MB)

ESM 2 (DTD 45.1 KB)

## Data Availability

The data that support the findings of this study are openly available in zenodo.org at 10.5281/zenodo.17866132.
