# Supplementary material for: Compensating thickness effects in micro X-ray fluorescence spectroscopy using integrated optical microscopy for thickness determination of soft matter block copolymer membranes
Source: Anal Bioanal Chem. 2025 Dec 19;418(16):5159–70. doi: 10.1007/s00216-025-06292-2 (PMC13424536; doi:10.1007/s00216-025-06292-2)
Supplement: Supplementary file 1 — (DOCX 2.75 MB) [file 216_2025_6292_MOESM1_ESM.docx]

# Supporting Information

**Compensating thickness effects in micro X-ray fluorescence spectroscopy using integrated optical microscopy for thickness determination of soft matter block copolymer membranes**

*Riccarda Müller^1^, Leon Weckenmann^1^, Nigar Aslanova^2^, Yesleen Gupta^3^, Felix Schacher ^2,4,5,6^, Carsten Streb^3^, Kerstin Leopold^1,^**

1) Institute of Analytical and Bioanalytical Chemistry (IABC), Ulm University, Albert-Einstein-Allee 11, Ulm 89081, Germany

2) Institute of Organic Chemistry and Macromolecular Chemistry (IOMC), Friedrich Schiller University Jena, Lessingstraße 8, 07743 Jena, Germany

3) Department of Chemistry, Johannes Gutenberg University Mainz, Duesbergweg 10-14, 55128, Mainz, Germany

4) Jena Center for Soft Matter (JCSM), Friedrich Schiller University Jena, Philosophenweg 7, 07743 Jena, Germany

5) Center for Energy and Environmental Chemistry Jena (CEEC Jena), Philosophenweg 7a, 07743 Jena, Germany

6) Helmholtz Institute for Polymers in Energy Applications Jena (HIPOLE Jena), Lessingstraße 12-14, 07743 Jena, Germany

* Corresponding author

Email: [kerstin.leopold@uni-ulm.de](mailto:kerstin.leopold@uni-ulm.de)

**Statistical calculations**

Quadratic error propagation was used to calculate statistical errors. For *f(x,y)* the quadratic error is calculated by the following equation:

$\Delta f\left( x,y \right)= \sqrt{\left( \frac{\partial f}{\partial x}\Delta x \right)^{2}+\left( \frac{\partial f}{\partial y}\Delta y \right)^{2}}$ *Equation (S1)*

The combined standard deviation *s* of a membrane thickness *d* at position *i* is calculated based on the standard deviations of the Z-coordinate of the empty measurement z*_0_* and the measurement of the membrane *z_i_*. Leading to the following equation:

$s\left( d_{i} \right)= \sqrt{s^{2}\left( z_{0} \right)+s^{2}(z_{i})}$ *Equation (S2)*

A modified *Welch’s* t test for unpaired samples according to *Linsinger et al.* [1] is used to determine the significant differences between two independent results $\bar{x_{1}} \pm s_{1}$ and $\bar{x_{2}} \pm s_{2}$. A normal distribution, a data set without significant outliers and a statistical confidence level of 95% are assumed. The *t* value is calculated according to the equation S3 with sample size *n*. As two results with $n_{1}=n_{2}=1$ are compared, the equation can be simplified using the absolute difference $\Delta_{m}$ and the combined uncertainty $u_{\Delta}$between the two values:

$t= \frac{\bar{x_{1}}-\bar{x_{2}}}{\sqrt{\frac{s_{1}^{2}}{n_{1}}+\frac{s_{2}^{2}}{n_{2}}}}= \frac{\bar{x_{1}}-\bar{x_{2}}}{\sqrt{s_{1}^{2}+s_{2}^{2}}}=\frac{\Delta_{m}}{u_{\Delta}}$ *Equation (S3)*

For *n = 1* the degrees of freedom are not defined because the denominator is zero. Therefore, a coverage factor or 2 is used for *P = 0.95*.

Paired *Student's* t-test is applied to calculate the significant difference between dependent samples. For the test normal distribution and no outliers is assumed. The *t* value is calculated using the mean value of differences $\bar{D}$, the standard deviation of differences $s_{D}$ and the number of pairs *N*.

$t= \frac{\bar{D}\sqrt{N}}{s_{D}}$ *Equation (S4)*

degrees of freedom *f* are determined according to:

$f=n-1$ *Equation (S5)*

**Total reflection X-ray fluorescence spectrometry (TXRF)**

To measure the elemental composition of the blue and silver ink the high-efficiency module S2 Picofox (Bruker Nano GmbH, Berlin, Germany) was used. The instrument is equipped with a Mo X-ray source and operated at maximum power conditions (50kV, 600 µA). The respective inks were applied to a precleaned and siliconized quartz glass sample carrier and measured with a live time of 1000s. Evaluation of the obtained spectra was achieved using Spectra PicoFox (7.2.5.0, Bruker Nano GmbH) software.

Table S1 Elemental composition of the inks used for autofocus markings as obtained by TXRF analysis

|  | ***Silver pen*** | ***Blue pen*** |
| --- | --- | --- |
| Main component(s) | Al | Cu, S, Cl |
| Traces | Fe, Ca, Zn, Ga, P, S, Cl ,K, Ti, Cr, Ni, Cu, Br | K, Ca, Fe, Br |

Table S2 Replicate autofocussing (n=10) for the blue mark on white paper with lighting intensity 30 a.u. in comparison to the blue mark on black paper with lighting intensity 11 a.u. and the silver mark on black paper with lighting intensity 30 a.u. in comparison to the silver mark on white paper with lighting intensity 30 a.u.

| ***(n = 10)*** | ***blue*** | | ***silver*** | |
| --- | --- | --- | --- | --- |
| ***Paper colour***  ***Lighting intensity*** | ***White***  ***30 a.u.*** | ***Black***  ***11 a.u.*** | ***Black***  ***30 a.u.*** | ***White***  ***30 a.u.*** |
| Grey value spread / a.u. | 46.67 ± 0.19 | 9.75 ± 0.01 | 94.52 ± 0.01 | 2.22 ± 0.01 |
| Std dev. Z-coordinate / µm | ± 30 | ± 7 | ± 3 | ± 6 |

Table S3 Thickness of five individual membrane pieces of Membrane A as determined by IOM stage method. Errors represent uncertainty as derived from Gaussian error propagation of replicate measurements with N = see table and P = 95%.

| ***Piece No.*** | ***Membrane thickness / µm*** | ***N*** |
| --- | --- | --- |
| 1 | 123 ± 7 | 36 |
| 2 | 111 ± 11 | 18 |
| 3 | 112 ± 8 | 18 |
| 4 | 157 ± 7 | 18 |
| 5 | 86 ± 17 | 9 |

Table S4 Thickness of Membrane D in µm determined when either the top or the bottom side of the membrane is facing upwards on two different measurement days. Errors represent uncertainty as derived from Gaussian error propagation of replicate measurements with N = 9 and P = 95%.

|  | ***Series 1: 0 days*** | ***Series 2: 11 days*** |
| --- | --- | --- |
| Piece 1 top side | 108 ± 14 | 79 ± 10 |
| Piece 1 bottom side | 99 ± 11 | 90 ± 11 |
| Piece 2 top side | 123 ± 15 | 122 ± 10 |
| Piece 2 bottom side | 138 ± 13 | 124 ± 9 |
| Piece 3 top side | 141 ± 16 | 128 ± 10 |
| Piece 3 bottom side | 115 ± 12 | 125 ± 12 |


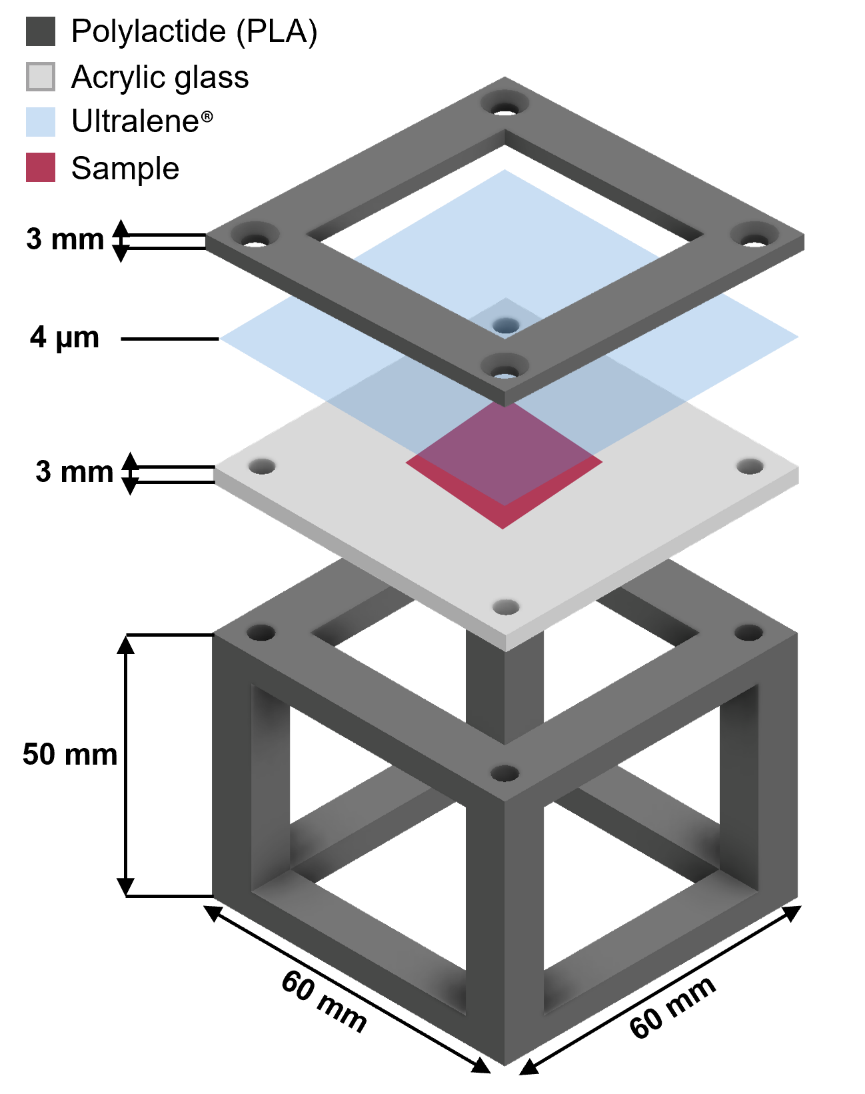


Figure S1 Exploded view of the 3D-printed sample holder for thickness measurements


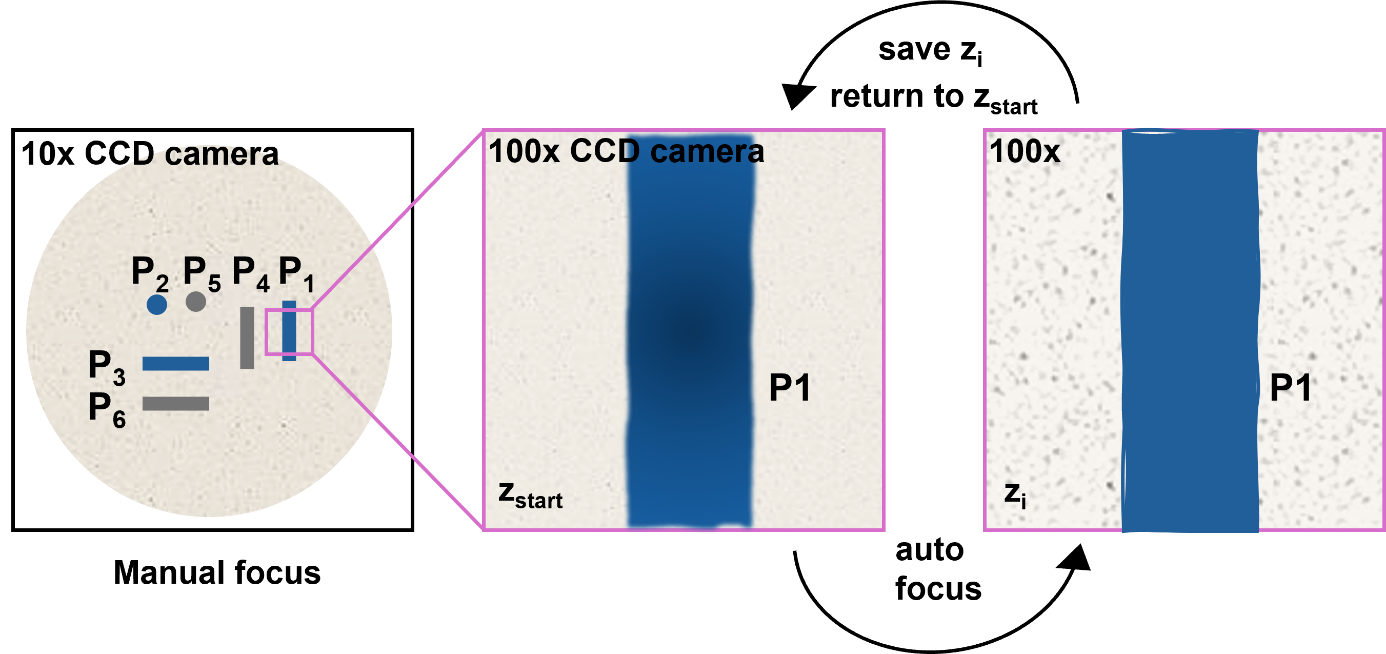


Figure S2 Schematic illustration of thickness measurement procedure

**Synthesis of block copolymer**

PS-*b*-PDMAEMA was synthesized in a two-step procedure *via* nitroxide-mediated polymerization (NMP), adapted from the literature [2]. In the first step, the hydrophobic block (PS) was obtained by NMP of styrene. In the second step, the polystyrene macroinitiator was used for the subsequent NMP of DMAEMA, yielding the final block copolymer PS-*b*-PDMAEMA. For the synthesis of the polystyrene (PS) macroinitiator, BlocBuilder MA (200 mg, 0.52 mmol) was dissolved in 60 mL styrene ([M]/[I] = 1000). Oxygen was removed from the reaction mixture by three freeze–pump–thaw cycles, and the reaction vessel was subsequently filled with argon. The mixture was heated at 110°C for 23 h and then cooled in liquid nitrogen. The resulting polymer was precipitated twice in cold methanol (Mₙ = 77,700 g mol⁻¹, Đ = 1.07).

In the second step, 1.5 g of the PS macroinitiator (Mₙ = 77,700 g mol⁻¹) was dissolved in 4.5 mL tetrahydrofuran (THF) together with DMAEMA (1.46 mL, [M]/[I] = 450). The stabilizer in DMAEMA was removed by passing the monomer through a short column of basic alumina prior to use. The reaction mixture was degassed by three freeze–pump–thaw cycles, filled with argon, and heated at 110°C for 1.9 h. After polymerization, the reaction mixture was cooled to room temperature and precipitated three times in cold hexane (Mₙ = 125,700 g mol⁻¹, Đ = 1.18). Characterization of the synthesized block copolymer by nuclear magnetic resonance (NMR) and size-exclusion chromatography (SEC) can be found in the SI (see Figure S3).


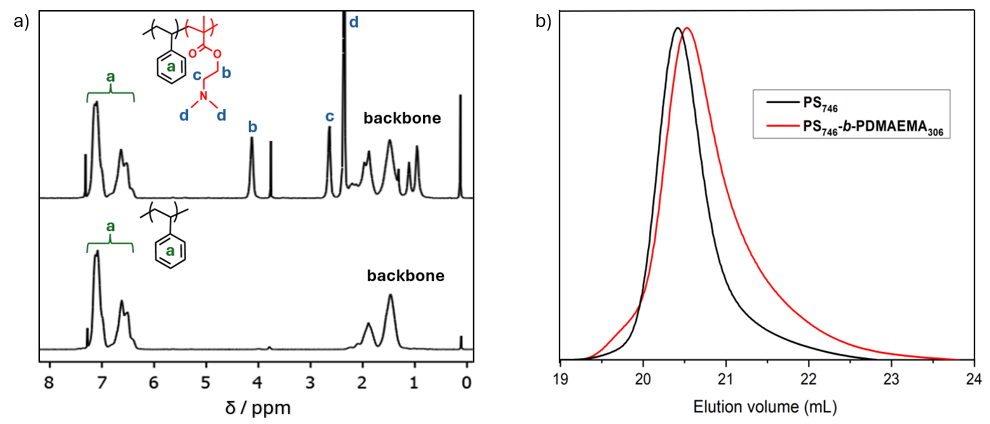


Figure S3a) NMR of PS-*block*-poly(N,N-dimethylaminoethyl methacrylate); b) SEC elution traces (THF, PS calibration) of PS (black) and PS-b-PDMAEMA (red).

**Membrane formation**

Porous block copolymer membranes were formed using the NIPS process [3]. Polymer solutions (15 wt%) were prepared in THF/DMF mixtures, with ratios of 70/30 wt% for more porous membranes and 30/70 wt% for less porous membranes. The solutions were cast onto clean glass substrates using a 200 μm doctor blade. After casting, the films were left for 30 s (“open time”) to allow partial solvent evaporation, followed by immersion in a water coagulation bath. After a few minutes, the membrane was lifted off the glass substrate and then stored in deionized water. All casting procedures were carried out in a *Plas-Labs* (US) glovebox under controlled humidity (50%) and temperature (24°C). Compositions of the membranes used in this work are summarised in Table S5. Two exemplary cross-sections with pore magnifications of the block copolymer membrane by scanning electron microscopy (SEM) are displayed in Figure S4:

Table S5 Composition of the used block copolymer membranes

| ***Membrane*** | ***Polymer Composition / repeated units*** | ***THF to DMF*** |
| --- | --- | --- |
| A | PS_746_-*b*-PDMAEMA_306_ (M1-M4)  PS_746_-*b*-PDMAEMA_284_ (M5) | 70/30 wt%  70/30 wt% |
| B | PS_671_-*b*-PDMAEMA_205_ | 70/30 wt% |
| C | PS_671_-*b*-PDMAEMA_205_ | 30/70 wt% |
| D | PS_654_-*b*-PDMAEMA_108_ | 70/30 wt% |
| E | PS_671_-*b*-PDMAEMA_205_ | 70/30 wt% |

**Synthesis of POM**

The polyoxometalate was prepared following the reported procedure [4]. Na_2_WO_4_·2H_2_O (35.5 g, 0.108 mol) and Co(NO_3_)_2_·6H_2_O (7.0 g, 0.025 mol) were dissolved in 100 mL water and stirred vigorously for 10 min before Na_2_HPO_4_·7H_2_O (3.22 g, 0.012 mol) was added. The pH of the resulting purple suspension was adjusted from 9 to 7 with 1M HCl and the mixture was then refluxed at 100 °C for 2 h. After reflux, the solution was saturated with NaCl (5-6 g) and cooled to room temperature. Purple crystals of [Co_4_(H_2_O)_2_(PW_9_O_34_)_2_]^10-^ were formed, which were collected by filtration, washed with water (30 mL) and recrystallized from hot water to afford the pure compound. (40% yield based on Co).


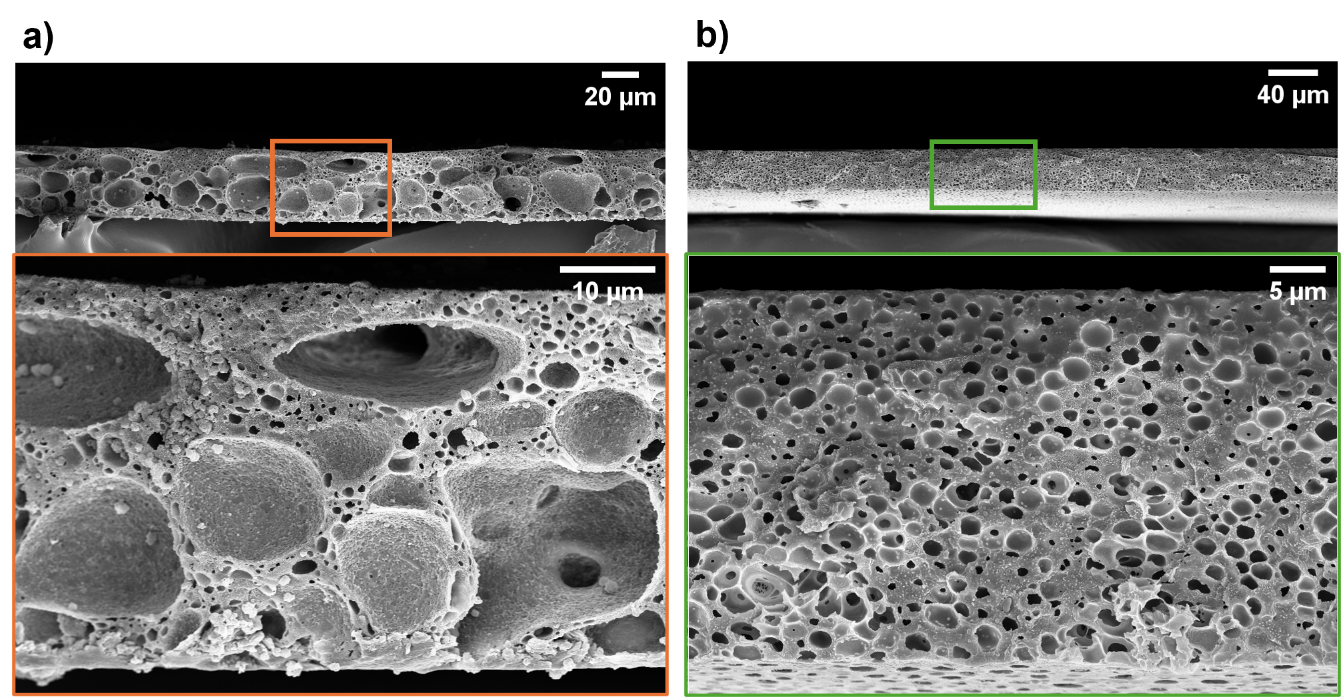


**Figure S4** Exemplary cross-sections and corresponding pore magnifications of dried and Pd-coated nanoporous block copolymer membranes using scanning electron microscopy.


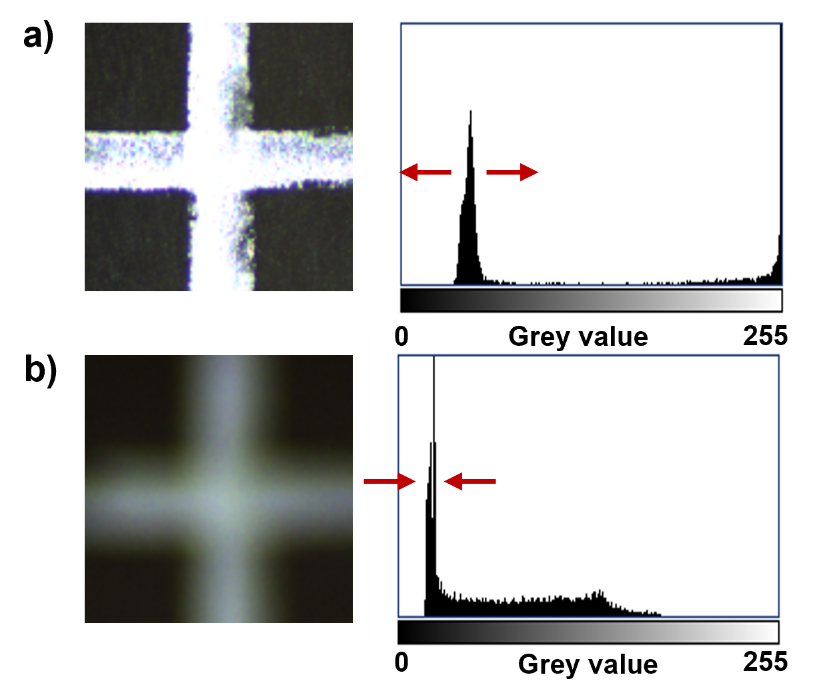


Figure S5 Comparison of the grey value spread of a) a sharp and b) a blurred image of a silver mark on an exemplary simulated sample taken by using the CCD camera at 10 times magnification


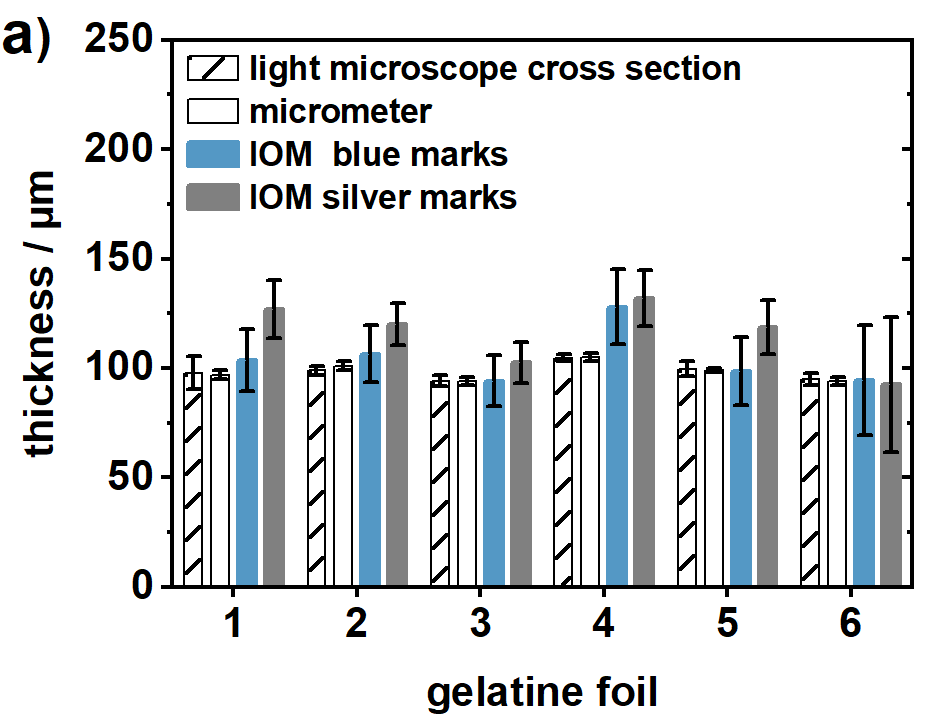

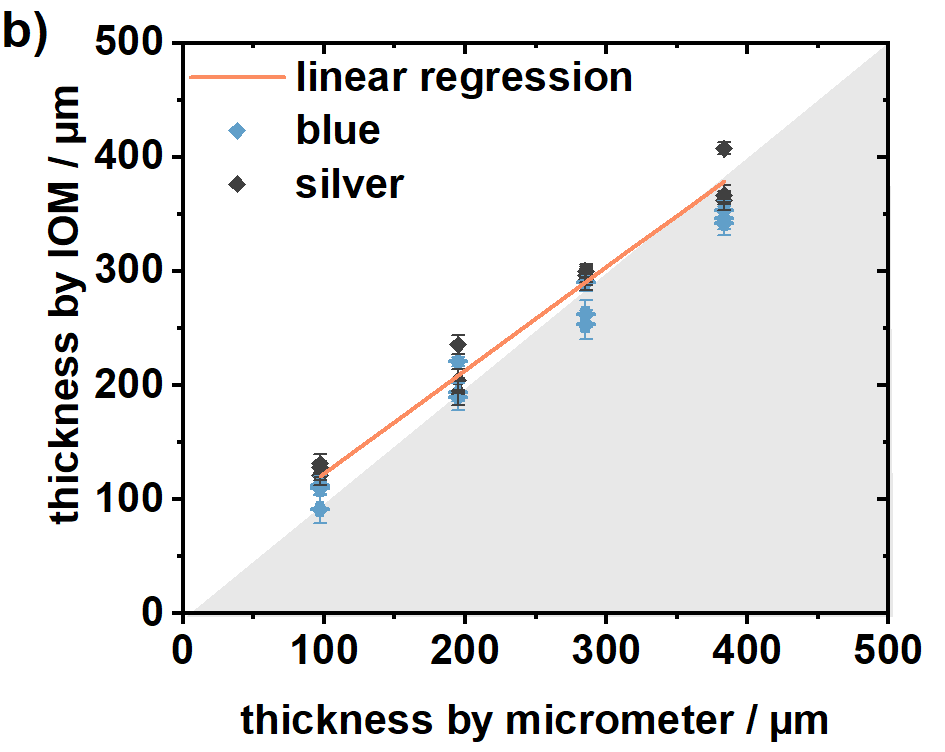


Figure S6a) Comparison of obtained thickness of six individual gelatine film samples as determined using a light microscopy at a cross-section (white dashed bars) and micrometre (white plain bars) as reference methods and IOM stage method using blue (blue bars) and silver (grey bars) marks.
b) Recovery plot for thickness determination of stacked gelatine films using IOM stage method with blue and silver marking vs. micrometre. Recovery function: $\boldsymbol{y =}\left( \boldsymbol{0.90 \pm0.04} \right)\boldsymbol{x+ (32.3 \pm9.63)}$. (N= 24; P=95%). Error bars reflect combined standard deviations of replicate measurements (n=3).


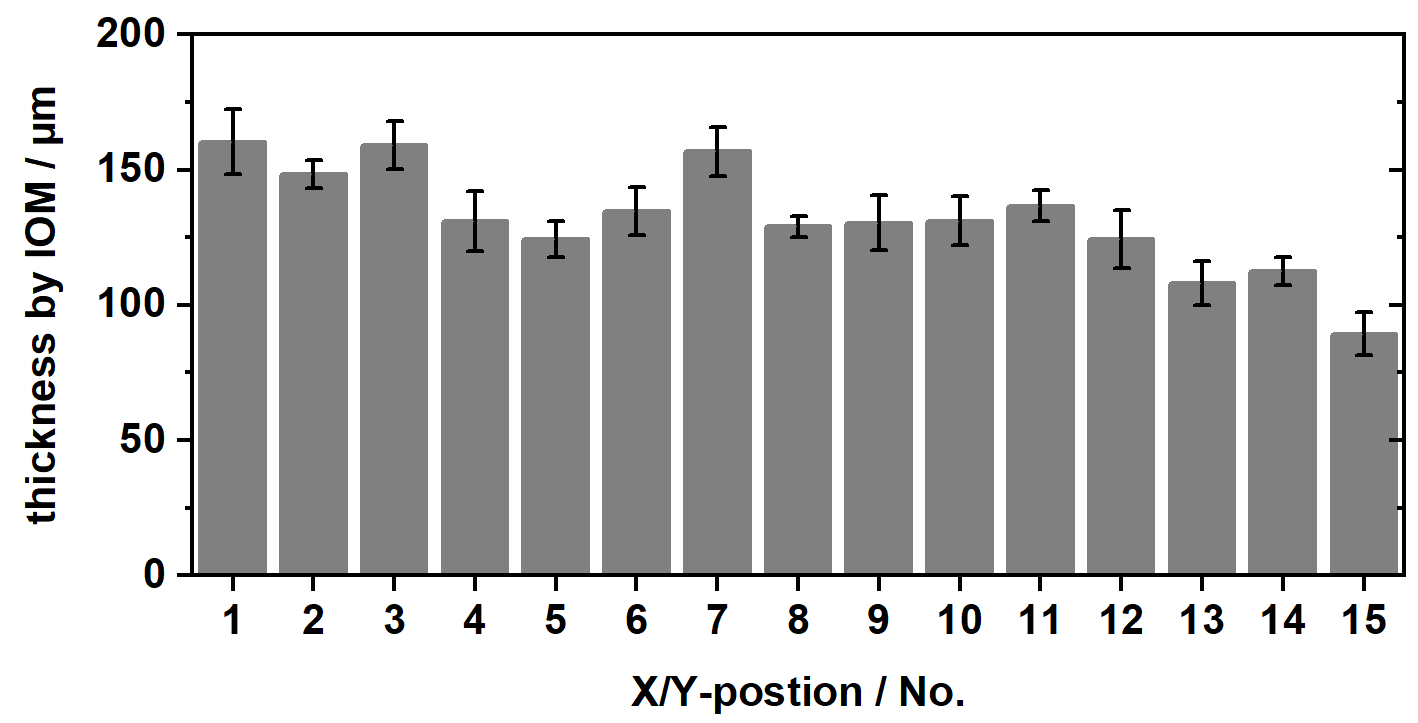


Figure S7 Thicknesses determined by IOM stage method at 15 randomly selected x/y-positions on *Membrane B*


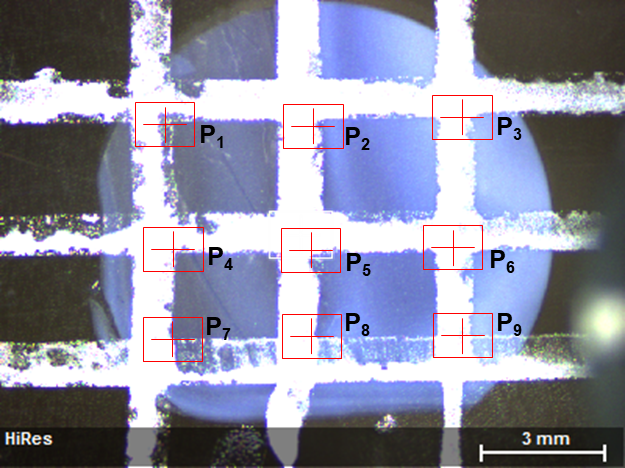


Figure S8 Photo of *Membrane E* with checkered silver marking on the cover film and marked positions


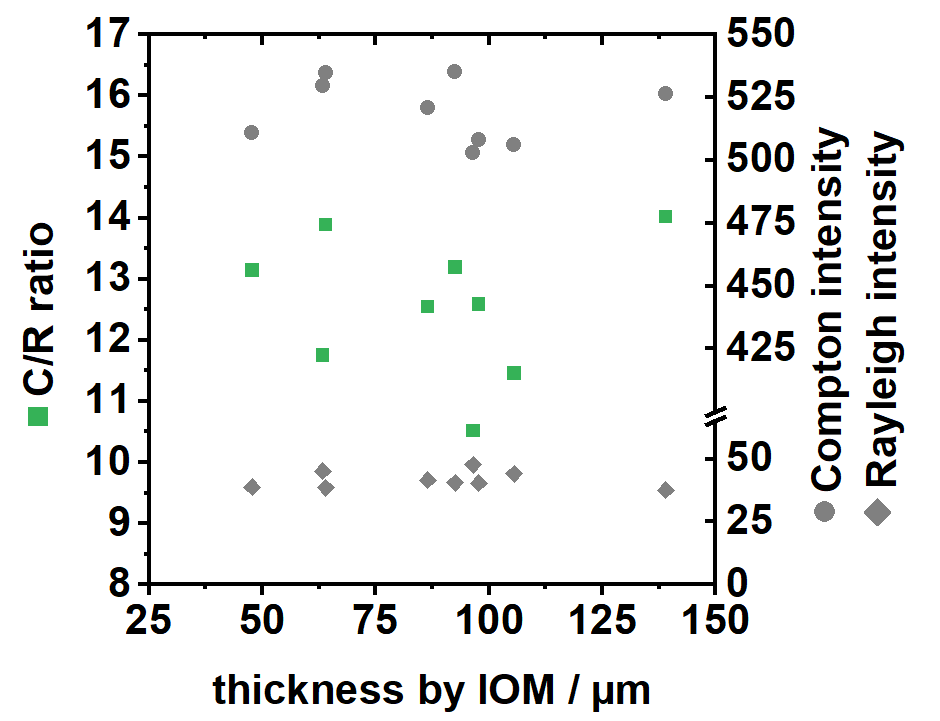


Figure S9 Intensities of scattering peaks (*Compton*: circles; *Rayleigh*: diamonds) and C/R ratio (squares) detected at the nine x/y-positions.

**References**

[1] T. Linsinger, ed., Guide to the expression of uncertainty in measurement, 1st ed. 1993, International Organization for Standardization, Genève, Switzerland, 1993.

[2] J. Kund, J. Kruse, A. Gruber, I. Trentin, M. Langer, C. Read, G. Neusser, D. Blaimer, U. Rupp, C. Streb, K. Leopold, F.H. Schacher, C. Kranz, Multimodal Analysis of Light‐Driven Water Oxidation in Nanoporous Block Copolymer Membranes**, Angew Chem Int Ed 62 (2023) e202217196. https://doi.org/10.1002/anie.202217196.

[3] I. Romanenko, M. Lechner, F. Wendler, C. Hörenz, C. Streb, F.H. Schacher, POMbranes: polyoxometalate-functionalized block copolymer membranes for oxidation catalysis, J. Mater. Chem. A 5 (2017) 15789–15796. https://doi.org/10.1039/C7TA03220J.

[4] Q. Yin, J.M. Tan, C. Besson, Y.V. Geletii, D.G. Musaev, A.E. Kuznetsov, Z. Luo, K.I. Hardcastle, C.L. Hill, A Fast Soluble Carbon-Free Molecular Water Oxidation Catalyst Based on Abundant Metals, Science 328 (2010) 342–345. https://doi.org/10.1126/science.1185372.
